# Noncontiguous finished genome sequence and description of *Intestinimonas massilien*sis sp. nov strain GD2^T^, the second *Intestinimonas* species cultured from the human gut

**DOI:** 10.1002/mbo3.621

**Published:** 2018-04-14

**Authors:** Pamela Afouda, Guillaume A. Durand, Jean‐Christophe Lagier, Noémie Labas, Fréderic Cadoret, Nicholas Armstrong, Didier Raoult, Grégory Dubourg

**Affiliations:** ^1^ Microbes, Evolution, Phylogeny and Infection Aix‐Marseille Université UM 63 CNRS 7278 IRD 198 Inserm 1095 IHU ‐ Méditerranée Infection 19‐21 Boulevard Jean Moulin 13005 Marseille France

**Keywords:** anaerobe, butyrate, culturomics, new species, taxono‐genomics

## Abstract

*Intestinimonas massiliensis* sp. nov strain GD2^T^ is a new species of the genus *Intestinimonas* (the second, following *Intestinimonas butyriciproducens* gen. nov., sp. nov). First isolated from the gut microbiota of a healthy subject of French origin using a culturomics approach combined with taxono‐genomics, it is strictly anaerobic, nonspore‐forming, rod‐shaped, with catalase‐ and oxidase‐negative reactions. Its growth was observed after preincubation in an anaerobic blood culture enriched with sheep blood (5%) and rumen fluid (5%), incubated at 37°C. Its phenotypic and genotypic descriptions are presented in this paper with a full annotation of its genome sequence. This genome consists of 3,104,261 bp in length and contains 3,074 predicted genes, including 3,012 protein‐coding genes and 62 RNA‐coding genes. Strain GD2^T^ significantly produces butyrate and is frequently found among available 16S rRNA gene amplicon datasets, which leads consideration of *Intestinimonas massiliensis* as an important human gut commensal.

## 1 INTRODUCTION

The description of the human microbiome has become one the most exciting challenges of the 21st century in the field of microbiology, as reflected by the Human Microbiome Project (HMP) (Turnbaugh et al., 2007). In particular, alterations in the composition of the human gut microbiota have been associated with several diseases, including obesity and inflammatory bowel disease. More recently, specific microbial signatures were predictive of the response to anticancer therapy in lung cancer (Vétizou et al., 2015). While high‐throughput sequencing techniques have enabled substantial advances in understanding the role exerted by the gut microbiota in human health, several limitations of these methods have been extensively discussed (Poretsky, Rodriguez‐R, Luo, Tsementzi, & Konstantinidis, 2014). Among these, 16S rRNA gene sequences may not match to a corresponding species in the database, which can potentially lead to missed and unknown taxa of great interest. Recently, Lagier et al. (2012, 2016) have shown that extensive bacterial culture, referred to as culturomics, can fill in the blanks of metagenomic data through the discovery of hundreds of new bacterial species associated with humans.

Considering the limitations of the traditional combination of phenotypic and genotypic characteristics to describe these new species (Kim, Oh, Park, & Chun, 2014; Rosselló‐Mora, 2006; Tindall, Rosselló‐Móra, Busse, Ludwig, & Kämpfer, 2010; Wayne et al., 1987), we also proposed using genomic information to help define and describe new bacterial species (Fournier, Lagier, Dubourg, & Raoult, 2015). We isolated a species belonging to the *Intestinimonas* genus as part of a culturomics study, using an anaerobic culture applied to a stool sample from a healthy subject. The genus *Intestinimonas,* which belongs to the *Firmicutes* phylum, was created in 2013. To date, this genus contains only *Intestinimonas butyriciproducens* gen. nov, sp. nov, which was first isolated from mice (Kläring et al., 2013). It has also been cultured from the human gut (Bui et al., 2015). Furthermore, it has recently been abundantly detected in human colonic samples (Bui et al., 2015), with a particular focus on butyrate production. In this paper, we present a summary of the classification and set of features for *Intestinimonas massiliensis* sp. nov. strain GD2^T^, together with a description of its complete genomic sequencing and annotation. These characteristics enable the creation of the *Intestinimonas massiliensis* species, which represents the second *Intestinimonas* species and the first cultured from the human gut microbiota.

## 2 MATERIAL AND METHODS

### 2.1 Sample information

The specimen was sampled from a healthy 28‐year‐old male of French origin, with a body mass index of 23.4 kg/m^2^. Consent was obtained, and the study was approved by the Institut Fédératif de Recherche 48 (Faculty of Medicine, Marseille, France), under agreement Number 09‐022.

### 2.2 *Strain identification and phylogenetic classification*

Strain GD2^T^ was isolated in February 2015 from a stool stored 10 days at −20°C after preincubation 72 hr and subculture under strict anaerobic conditions in the presence of sheep blood (5%) and rumen fluid (5%). Identification was performed using MALDI‐TOF mass spectrometry and by sequencing of the 16S rRNA gene. DNA extraction was realized using an EZ1 DNA Tissue Kit (Qiagen, Courtaboeuf, France). The DNA extract was amplified using PCR technology and universal primers FD1 and RP2 (Eurogentec, Angers, France). The amplifications and sequencing of the amplified products were performed as previously described (Dubourg et al., 2013). Afterward, 16S rRNA gene sequences were compared with those available in GenBank (http://www.ncbi.nlm.nih.gov/genbank/). When the percentage of identity of the entire 16S sequence was below the generally accepted threshold of 98.65%, the studied strain was considered as a new species (Kim et al., 2014).

Phylogenetic analysis based on 16S rRNA of our isolate was performed to identify its phylogenetic affiliations with other near isolates, including other members of the genus *Intestinimonas*. The MEGA 6 (Molecular Evolutionary Genetics Analysis) software enabled us to build a phylogenetic tree (Tamura, Stecher, Peterson, Filipski, & Kumar, 2013). The use of CLUSTALW permitted us to align the sequences of different species (Thompson, Higgins, & Gibson, 1994) and the Kimura two‐parameter model was used to calculate evolutionary distance (Kimura, 1980).

### 2.3 Physiological and phenotypic characteristics

The strain was tested for growth in anaerobic conditions at varying temperatures: 28°C, 37°C, 45°C, and 56°C. Growth under aerobic and microaerophilic conditions was also assessed. To determine the biochemical characteristics of the strain, API ZYM (bioMérieux), API Rapid ID 20 NE (bioMérieux), and API 50 CH (bioMérieux) were used, following the instructions of the manufacturer. Catalase and oxidase activities were also tested. Gram staining and motility were determined using the light microscope DM1000 (Leica Microsystems, Nanterre, France). Cell morphology was determined using Tecnai G20 transmission electron microscopy (FEI Company, Limeil‐Brévannes, France), after negative staining of the bacteria and elements determining the gram‐stain characteristics of the bacteria were evaluated using the Morgagni 268D TEM (Philips). For preparation for transmission electron microscopy (TEM), bacteria were recovered and pelleted for 10 min at 5,000 g. The pellet was resuspended in 1 ml of phosphate‐buffered saline (PBS) with 2.5% glutaraldehyde in a 0.1mol/L sodium cacodylate buffer and incubated for at least 1 hr at 4°C. The pellet was then washed three times with 0.1mol/L cacodylate‐saccharose and resuspended in the same buffer. After repelleting, the sample was embedded in Epon resin using a standard method, as follows: 1 hr of fixation in 1% osmium tetroxide, two washes in distilled water, dehydration in increasing ethanol concentrations (30%, 50%, 70%, 96%, and 100% ethanol), and embedding in Epon‐812. Ultrathin sections of 70 nm were poststained with 5% uranyl acetate and lead citrate following the Reynolds method (Reynolds, 1963). Samples were then observed using a Morgagni 268D TEM (Philips) operating at 60 keV. To determine sporulation, thermal shock was carried out on the bacteria at 80°C for 20 min, which were then seeded on Colombia blood agar. Plates were then incubated for 48 hr under anaerobic conditions. We determined antibiotic susceptibility using the E‐test gradient strip method (bioMérieux) to define the minimal inhibitory concentration (MIC) of each tested antibiotic. After culture of strain GD2^T^ on 5% sheep blood‐enriched Columbia agar (bioMérieux), the bacterial inoculum of 0.5 McFarland turbidity was prepared by suspending the culture in sterile saline (0.85% NaCl). Due to the inability of *Intestinimonas massiliensis* to grow on the medium recommended by EUCAST (Citron, Ostovari, Karlsson, & Goldstein, 1991; Matuschek, Brown, & Kahlmeter, 2014) (i.e., MH‐F agar), the bacterial suspension was swabbed on Columbia agar (bioMérieux). Then, each of the E‐test strips (amoxicillin, ceftriaxone, ofloxacin, penicillin G, imipenem, and vancomycin) were separately placed in culture plates and incubated under anaerobic conditions for 72 hr. The test was done in duplicate and a quality control was done with the *Escherichia coli* strain DSM 1103. The MIC was determined by measuring the intersection of the E‐test strips with the elliptic zones of inhibition (Citron et al., 1991).

### 2.4 Fatty acid methyl ester analysis

Cellular fatty acid methyl ester (FAME) analyses of *Intestinimonas massiliensis* strain GD2^T^ (=CSUR P1930) and *Intestinimonas butyriciproducens* (=CSUR P1453 = DSM 103501) were performed using GC/MS. Two bacterial biomass sample tubes of about 4 mg each obtained from cultures plates were prepared after 72 hr of culture of the bacteria on 5% sheep blood‐enriched Columbia agar (bioMérieux) in anaerobic conditions. Then, fatty acid methyl esters were prepared according to the description of Sasser (2006). GC/MS analyses were carried out as previously stated (Dione et al., 2016). Mass spectrometry (Clarus 500 ‐ SQ 8 S, Perkin Elmer, Courtaboeuf, France) allowed us to separate fatty acid methyl esters by utilization of an Elite 5‐MS column. Utilization of the Standard Reference Database 1A (NIST, Gaithersburg, USA) and the FAMEs mass spectral database (Wiley, Chichester, UK), permitted us to search a spectral database with MS Search 2.0.

### 2.5 Short‐chain fatty acids analysis

Short‐chain fatty acids (SCFA) were measured with a Clarus 500 chromatography system connected to a SQ8s mass spectrometer (Perkin Elmer, Courtaboeuf, France), as previously detailed (Zhao, Nyman, & Åke, 2006), with modifications. As a prelude to this, 500 μg of bacterial suspension were placed in Lytic/10 anaerobic/F (BD ^™^ Bactec ^™^ Media) medium and incubated at 37°C for 72 hr. Acetic, propanoic, isobutanoic, butanoic, isopentanoic, pentanoic, hexanoic, and heptanoic acids were purchased from Sigma Aldrich (Lyon, France). A stock solution was prepared in water/methanol (50% v/v) at a final concentration of 50 mmol/L and then stored at −20°C. Calibration standards were freshly prepared in acidified water (pH 2‐3 with HCl 37%) from the stock solution at the following concentrations: 0.5; 1; 5; 10 mmol/L. SCFA were analyzed from three independent culture bottles (both blanks and samples). Culture medium was collected, then centrifuged for 5 min at 16,000 g to remove bacteria and debris. The clear supernatant was adjusted to pH 2‐3 and spiked with 2‐ethylbutyric acid as the internal standard (IS) at a final concentration of 1 mmol/L (Sigma Aldrich). The solution was once again centrifuged before injection. Aqueous samples were directly injected (0.5 μl) in a splitless liner heated at 200°C. The injection carry‐over was decreased with 10 syringe washes in water/methanol (50:50 v/v). Compounds were then separated on an Elite‐FFAP column (30 m, 0.25 mm id 0.25 mm film thickness) using a linear temperature gradient from 100°C to 200°C at 8°C/min. Helium at a flow rate of 1 mL/min was utilized. The MS inlet line and electron ionization source were set at 200°C. To insure compound selectivity, selected ion recording (SIR) was performed after a 4.5 min solvent delay with the following masses: 43 m/z (isobutanoic acid), 60 m/z (acetic, butanoic, pentanoic, isopentanoic, hexanoic, and heptanoic acids) 74 m/z (propanoic acid), 88 m/z (2‐ethylbutyric acid, IS). All data were collected and processed using TurboMass 6.1 (Perkin Elmer, Courtaboeuf, France). Quadratic internal calibration was calculated for each acid using the peak areas from the associated SIR chromatograms. Coefficients of determination were all above 0.999. Back‐calculated standards and calculated quality controls (0.5 and 5 mmol/L) all showed good accuracy, with deviations below 15%. SCFA quantities in samples were presented after subtraction of the quantities measured in the blank samples.

### 2.6 Genomic sequencing

We used MiSeq Technology (Illumina Inc, San Diego, CA, USA) to sequence genome DNA (gDNA) of the *Intestinimonas massiliensis* strain GD2^T^, along with the mate pair strategy by Nextera Mate Pair sample prep kit (Illumina), as previously described (Lagier et al., 2014).

Using a Qubit assay with broad range kit (Life Technologies, Carlsbad, CA, USA) allowed to us to quantify genomic DNA to 137 ng/μl. Then, we prepared a mate pair library with 1.5 μg of gDNA using the Nextera mate pair Illumina guideline as per manufacturer's instructions. Afterward, we simultaneously splintered and tagged the gDNA sample with a mate pair junction adapter. Subsequently, to validate the splitting pattern, we used a DNA 7500 LabChip on the Agilent 2100 Bioanalyzer (Agilent Technologies Inc, Santa Clara, CA, USA). The fragments obtained had the required size of 6.01 kb. No size selection was performed, and tagged fragments 428.4 ng were circularized. Next, small fragments were obtained by mechanical shearing from the circularized DNA on the Covaris device S2 in T6 tubes (Covaris, Woburn, MA, USA). The optimal size of these small fragments was 950 bp. Following visualization of the library profile on the High Sensitivity Bioanalyzer LabChip (Agilent Technologies Inc, Santa Clara, CA, USA), the final concentration library obtained was 4.593 nmol/L.

This library was then combined with the other 11 projects and finally normalized to 2 nmol/L, which was further denatured and diluted to 15 pM. The automated cluster was generated after loading in the reactant cartridge along with the flow cell instrument, and a 39‐hour long sequencing run was carried out.

With a cluster density of 653 K/mm^2^, the information acquired represented a total of 6.1 Gb; this contains a group pass quality control filter estimated at 96.1% (12,031,000 pairs of pass filters). The index representation of the *Intestinimonas massiliensis,* corresponding to the proportion of reads attributed to this project among the total number of number of reads‐, was of 8.06%. The 1,208,418 paired reads were trimmed and afterward assembled into seven scaffolds.

### 2.7 Genome annotation and comparison

We predicted open reading frames (ORFs) utilizing Prodigal with default settings (http://prodigal.ornl.gov/) (Hyatt et al., 2010). All predicted ORFs not covering a region of the standard sequence were excluded. We searched predicted bacterial protein sequences against GenBank and Clusters of Orthologous Groups (COG) (Benson et al., 2012) using BLASTP. We then used the tRNAScan‐SE, RNAmmer tools (Lagesen et al., 2007; Lowe & Eddy, 1997), SignalP and TMHMM, (Bendtsen, Nielsen, Heijne, & Brunak, 2004; Krogh, Larsson, von Heijne, & Sonnhammer, 2001) for prediction of tRNAs, rRNAs, signal peptides, and numbers of transmembrane helices, respectively. PHAST and RAST were used to predict mobile genetic elements (Aziz et al., 2008; Zhou, Liang, Lynch, Dennis, & Wishart, 2011). Identification of ORF without homologues in other lineages (ORFans) depended on parameter thresholds of their BLASTP E‐value. So, identification was possible for BLASTP E‐values lower than 1e^−03^ for an alignment length greater than 80 amino acids, but if they were smaller than 80 amino acids we used an E‐value of 1e^−5^. Data management, visualization of genomic characteristics, and multiple genomic sequence alignment were performed by utilization of the Artemis DNAPlotter (Carver, Thomson, Bleasby, Berriman, & Parkhill, 2009; Rutherford et al., 2000) and alignment tools (version 2.3.1), respectively (Darling, Mau, Blattner, & Perna, 2004).

We took the complete sequence of the genome, the genome sequence of the proteome and genome sequence of the ORFeome from the FTP of NCBI. Proteomes were analyzed using Proteinortho (Lechner et al., 2011). The average similarity of orthologous proteins was evaluated using the Average Genomic Identity Of gene Sequences (AGIOS) software (Ramasamy et al., 2014). This allowed us to compare the pairwise orthologous proteins in combination with the Proteinortho software (Lechner et al., 2011). The corresponding genes were recovered and the percentage nucleotide identity among ORF orthologs was calculated using the Needleman–Wunsch global alignment algorithm. Finally, the Multi‐Agent Software System DAGOBAH was used to achieve all annotation and comparison processes (Gouret et al., 2011), including Figenix libraries that provide pipeline analysis (Gouret et al., 2005).

The 16S rRNA sequence of *Intestinimonas massiliensis* strain GD2^T^ was compared to those of other close species belonging to the *Firmicutes* phylum, such as *Intestinimonas butyriciproducens, Pseudoflavonifractor capillosus, Oscillibacter valericigenes*,* Flavonifractor plautii*,* Clostridium cellulosi*,* Clostridium viride*,* Ethanoligenens harbinense*,* Clostridium leptum,* and *Eubacterium siraeum*.

We performed the gel view for protein profile comparisons for *Intestinimonas massiliensis* strain GD2^T^ with the following Firmicutes species: *Intestinimonas butyriciproducens*,* Flavonifractor plautii*,* Clostridium papyrosolvens*, and *Clostridium cellobioparum*.

### 2.8 Frequency and relative abundance of Intestinimonas species among 16S rRNA sequence databases

To investigate the relative abundance and frequency of *I. massiliensis* and *I. butyriciproducens* we used the IMNGS open resource platform that provides a research of abundance of our 16S rRNA sequence into 16S rRNA gene amplicon datasets from the Sequence Read Archive (Lagkouvardos et al., 2016). For this purpose, the entire sequence of *I. massiliensis* (Genbank accession number LN866996) and that of *I. butyriciproducens* (Genbank accession number KC311367) was used for search with a similarity threshold of 99% and a minimum size of 200 bp. Results were then manually filtered, and frequency was calculated according to the origin of the sample. Samples for which only one sequence was detected were not considered as positive. To assess relative abundance of these two species in the human gut, only datasets labeled “human gut metagenome” were considered. Number of sequences attributed to the bacteria was divided by the sample size to estimate relative abundance.

## 3 RESULTS

### 3.1 Phylogenetic classification

**Figure 1:**
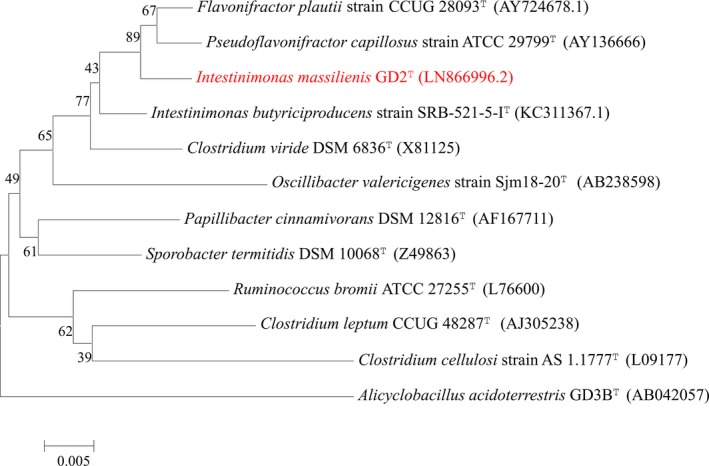
Phylogenetic tree highlighting the position of *Intestinimonas massiliensis* strain GD2 ^T^ relative to other Firmicutes. Numbers at the nodes are percentages of bootstrap values obtained by repeating the analysis 1,000 times to generate a majority consensus tree. The scale bar represents a 5% nucleotide sequence divergence

*Intestinimonas massiliensis* strain GD2^T^ was first isolated in February 2015 on agar enriched with sheep blood (5%) and rumen fluid (5%) at 37°C under anaerobic conditions (Table 1). The MALDI‐TOF MS spectrum was subsequently added to our database (Figure S1). The gel view highlights marked spectral differences with other members of the *Firmicutes* phylum (Figure S2), in particular with the *Intestinimonas butyriciproducens* spectrum.Identification of our strain by MALDI‐TOF yielded no reliable identification despite regular database updates. Strain GD2^T^ exhibited a 94.96% 16S rRNA sequence identity with the type strain *Intestinimonas butyriciproducens* SRB‐521‐5‐I^T^ (GenBank accession number KC311367), the phylogenetically closest bacterial species with standing in the nomenclature (Figure 1). Its 16S rRNA sequence was deposited in GenBank under number LN866996. This value was lower than the 98.65% 16S rRNA gene sequence threshold recommended by Kim et al. (2014) to delineate a new species without carrying out DNA–DNA hybridization.

**Table 1:** Classification and General Features of *Intestinimonas massiliensis* strains GD2^T^

Property	Term
Current classification	Domain: *Bacteria* Phylum: *Firmicutes* Class: *Clostridia* Order: *Clostridiales* Family: unclassified *clostridiales* Genus: *Intestinimonas* Species: *Intestinimonas massiliensis* Type strain: strain GD2^T^
Cell shape	Rod
Temperature range	Mesophilic
Optimum temperature	37°C
pH	6‐8.5
Salinity	0‐5 g/L

### 3.2 Phenotypic description

**Figure 2:**
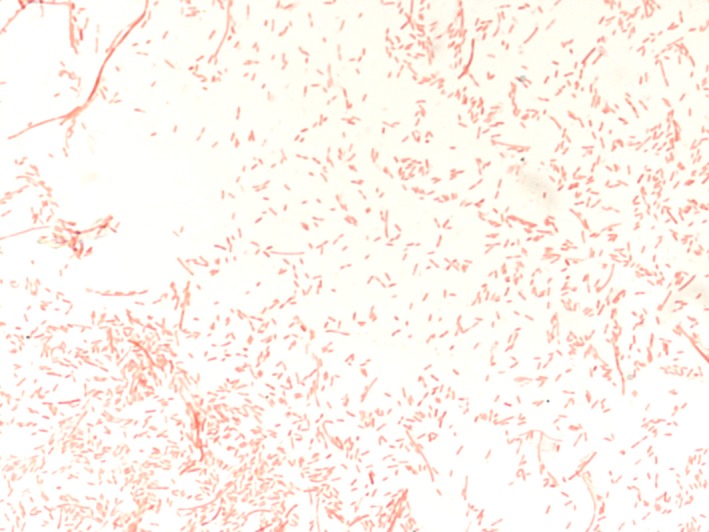
Gram staining of the *Intestinimonas massiliensis* strain GD2^T^

The growth of *Intestinimonas massiliensis* strain GD2^T^ was observed at 37°C after 72 hr of incubation in anaerobic conditions, whereas no growth was observed at 28°C, 45°C, and 56°C. No growth occurred under aerobic conditions. The *Intestinimonas massiliensis* strain GD2^T^ is thus strictly anaerobic and grows up to 37°C. Its pH range for growth was 6‐8.5 and it tolerated NaCl concentrations ranging from 0 to 5 g/L. Cells were immotile and nonsporulating. Colonies were regular, white, with a mean diameter of 1–2 mm on sheep blood‐enriched Colombia agar. Gram staining (Figure 2) showed gram‐negative rods. Using electron microscopy, the rods had a mean diameter of 0.5 μm and a length of 1.8 μm (Figure S3). Catalase and oxidase activities were negative for *Intestinimonas massiliensis* strain GD2^T^. Using API ZYM, positive reactions were observed for naphthol‐AS‐BI‐phosphohydrolase and acid phosphatase. Negative reactions were observed for alkaline phosphatase, esterase (C4), esterase lipase (C8), lipase (C14), leucine arylamidase, valine arylamidase, trypsin, α‐chymotrypsin, β‐galactosidase, N‐acetyl‐ β‐glucosaminidase, α‐galactosidase, β‐glucuronidase, α‐glucosidase, β‐glucosidase, α‐fucosidase, and α‐mannosidase.

An API 50 CH strip showed positive fermentation reactions for D‐arabinose, D‐ribose, D‐xylose, L‐xylose, D‐galactose, L‐sorbose, amygdalin, esculin ferric citrate, D‐melibiose, D‐trehalose, inulin, D‐melezitose, D‐raffinose, starch, glycogen, xylitol, gentiobiose, D‐lyxose, D‐tagatose, D‐fucose, and potassium 5‐ketogluconate, but a faint positive reaction was observed for D‐fructose. Negative fermentation reactions were recorded for glycerol, erythritol, L‐arabinose, D‐adonitol, methyl‐βD‐xylopyranoside, D‐glucose, D‐mannose, L‐rhamnose, dulcitol, inositol, D‐mannitol, D‐sorbitol, methyl‐αD‐mannopyranoside, methyl‐αD‐glucopyranoside, N‐acetyl‐glucosamine, arbutin, salicin, D‐cellobiose, D‐maltose, D‐lactose, D‐sucrose, D‐turanose, L‐fucose, D‐arabitol, L‐arabitol, potassium gluconate, and potassium 2‐ketogluconate. Using API 20 NE demonstrated a positive reaction for gelatin hydrolysis, but negative reactions for β‐galactosidase, potassium nitrate (nitrate reductase), L‐tryptophan (indole formation), D‐glucose (fermentation and assimilation), L‐arginine, urease, esculin ferric citrate, and negative assimilation reactions for L‐arabinose, D‐mannose, D‐mannitol, N‐acetylglucosamine, D‐maltose, potassium gluconate, capric acid, adipic acid, malic acid, trisodium citrate, and phenylacetic acid.

When compared with its phylogenetically closest neighbor (i.e., *Intestinimonas butyriciproducens* strain SRB‐521‐5‐I^T^), *Intestinimonas massiliensis* strain CD2^T^ differed in endospore formation, nitrate reductase, fermentation of L‐arabinose, and D‐glucose (Table 2).

**Table 2:** Differential characteristics of *Intestinimonas massiliensis* strain GD2^T^ **(1)** and other strains: *Intestinimonas butyriciproducens* SRB‐521‐5‐I^T^ **(2)**;* Flavonifractor plautii* DSM 4000^T^ **(3)**;* Pseudoflavonifractor capillosus* CCUG 15402A^T^ **(4);** and *Clostridium cellulosi* AS 1.1777 **(5)**. +, positive; ‐, negative; na, not available; V, variable; No., number

Properties	1	2	3	4	5
Cell diameter (μm)	1.8 × 0.5	2‐5	2‐7	na	0.3‐0.6
Oxygen requirement	strictly anaerobic	strictly anaerobic	strictly anaerobic	strictly anaerobic	strictly anaerobic
Gram stain	−	+	V	−	−
Motility	−	−	V	−	+
Endospore formation	−	+	V	−	+
Indole	−	Na	na	−	‐
Major Fatty acids					
FAME	16: 0; 18: 1n9	14: 0; 12: 0	16: 0; 14: 0	16: 0; 14: 0	na
DNA G+C content (mol %)	60.68	58.4	61.6	60	35
Genome size (bp)	3,104,261	3,376,475	3,818,478	4,241,076	5,680,000
Gene content (No.)	3,074	3,529	4,278	4,829	5,171
Production of					
Catalase	−	Na	na	−	−
Oxidase	−	Na	na	na	na
Nitrate reductase	−	+	na	na	−
Urease	−	−	na	na	na
β‐galactosidase	−	na	na	+	na
N‐acetyl‐glucosamine	−	na	na	na	na
Acid from					
L‐arabinose	−	+	+	na	−
D‐mannose	−	na	na	−	+
D‐mannitol	−	−	−	+	+
D‐glucose	−	+	−	+	+
D‐fructose	+/−	−	−	+	+
D‐maltose	−	−	−	+	+
D‐lactose	−	na	na	na	+
Habitat	Human gut	Mouse gut Human gut	Human gut	Human and animal gut	Cow manure compost
References	This study	(Bui et al., 2015; Kläring et al., 2013), This study	(Carlier, Bedora‐Faure, K'ouas, Alauzet, & Mory, 2010; Kläring et al., 2013), This study	(Kläring et al., 2013; Madsen & Justesen, 2011), This study	(He, Ding, & Long, 1991), This study

MICs for the GD2^T^ strain were distributed as follows: vancomycin (MIC 0.50 μg/ml), penicillin G (MIC 0.19 μg/ml), imipenem (MIC 0.25 μg/ml), ceftriaxone (MIC 1 μg/ml), and amoxicillin (MIC 0.125 μg/ml). A high level of resistance to ofloxacin was observed (MIC > 32 μg/ml).

### 3.3 Fatty acid methyl ester analysis

Cellular fatty acid composition showed that the two most abundant fatty acids are unsaturated 9‐octadecenoic acid (35%) and saturated hexadecanoic acid (30%) (Table 3). Table 3 also demonstrates the comparison of cellular fatty acid composition (%) of *Intestinimonas massiliensis* strain GD2^T^ with *Intestinimonas butyriciproducens* CSUR P1453‐DSM 103501; a significant difference is observed with 1 tetradecanoic acid, 2‐methyl‐tridecanoic acid, and hexadecanoic acid.

**Table 3:** Cellular fatty acid methyl ester composition (%) of *Intestinimonas massiliensis* strain GD2^T^

Fatty acids	IUPAC name	Mean Relative %^a^
12:0	Dodecanoic acid	TR
13:0	Tridecanoic acid	TR
14:0	Tetradecanoic acid	4.8 ± 1.1
14:0 iso	12‐methyl‐Tridecanoic acid	TR
15:0	Pentadecanoic acid	1.2 ± 0.1
15:0 iso	13‐methyl‐tetradecanoic acid	TR
15:0 anteiso	12‐methyl‐tetradecanoic acid	TR
16:0	Hexadecanoic acid	30.3 ± 5.5
16:0 9,10‐methylene	9,10‐methylene‐Hexanoic acid	TR
16:1n7	9‐Hexadecenoic acid	3.3 ± 0.2
17:0	Heptadecanoic acid	1.7 ± 1.4
17:1n7	10‐Heptadecenoic acid	1.4 ± 0.4
17:0 anteiso	14‐methyl‐Hexadecanoic acid	1.7 ± 0.3
18:0	Octadecanoic acid	7.7 ± 1.1
18:1n9	9‐Octadecenoic acid	34.6 ± 1.6
18:2n6	9,12‐Octadecadienoic acid	11.5 ± 2.8

a Mean peak area percentage ± standard deviation; TR: trace amounts < 1.

### 3.4 Short‐chain fatty acids analysis

Production of SCFA by *Intestinimonas massiliensis* strain GD2^T^ was positively detected, with a major production of butanoic acid (6.4 ± 0.7 mmol/L) and minor production of acetic (0.7 ± 0.1 mmol/L), propanoic (0.4 ± 0.4 mmol/L), and pentanoic (0.1 ± 0.1 mmol/L) acids. Isobutanoic, isopentanoic, hexanoic, and heptanoic acids were not produced.

### 3.5 Genome properties

**Figure 3:**
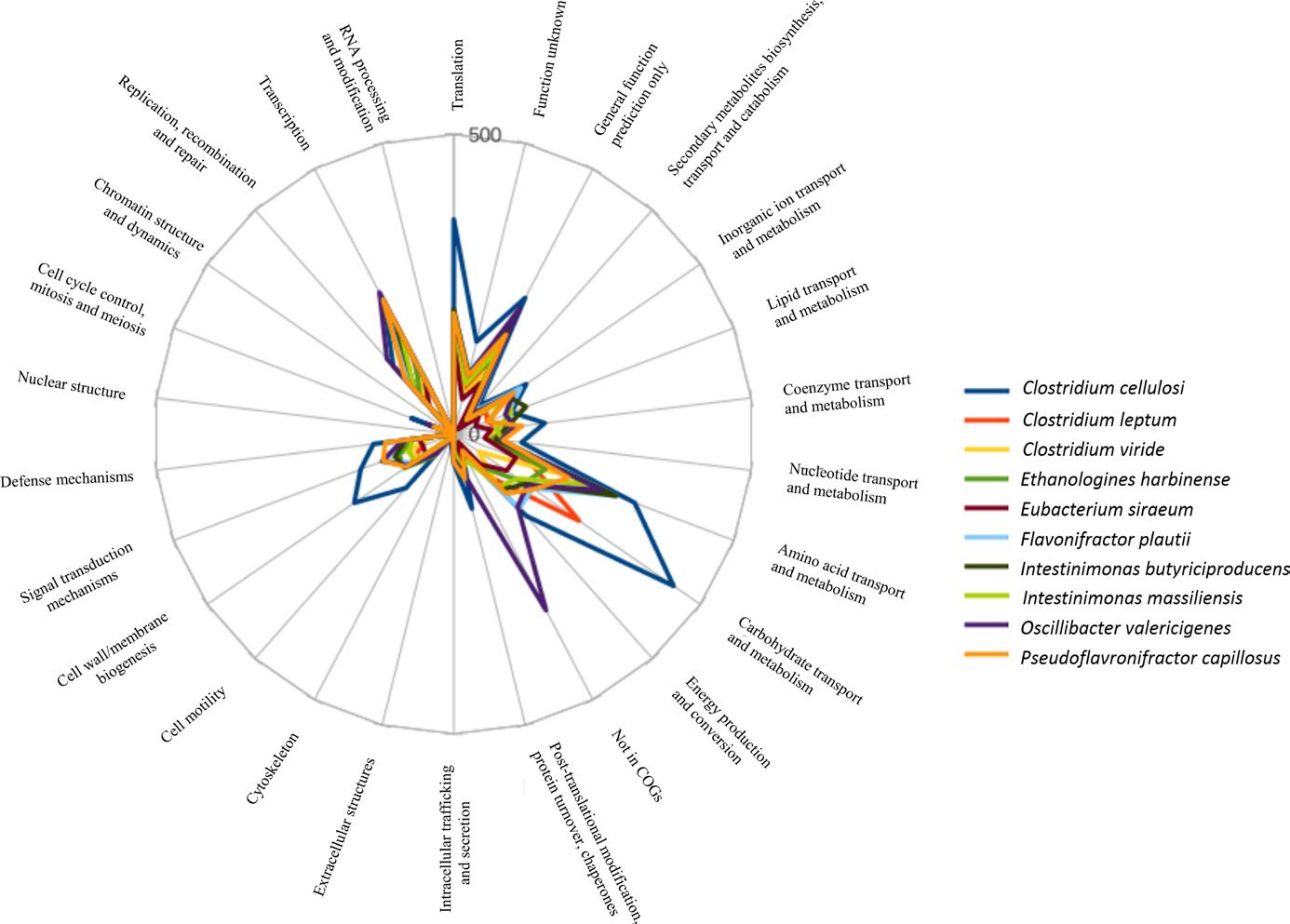
Distribution of functional classes of predicted genes on the chromosomes of strain CD2^T^ and related taxa *Clostridium cellulosi*,* Clostridium leptum*,* Clostridium viride*,* Ethanoligenens harbinense*,* Eubacterium siraeum*,* Flavonifractor plautii*,* Intestinimonas butyriciproducens*,* Intestinimonas massiliensis*,* Oscillibacter valericigenes*,* Pseudoflavonifractor capillosus*, according to the clusters of orthologous groups of protein

The genome of *Intestinimonas massiliensis* strain GD2^T^ is 3,104,261 bp long with 60.66% GC content. This noncontiguous finished genome is composed of seven scaffolds accounting for nine contigs. Of the 3,074 predicted genes, 3,012 were protein‐coding genes and 62 were RNAs (two genes were 5S rRNA, two genes were 16S rRNA, two genes were 23S rRNA, and 56 genes were TRNA genes). A total of 1,933 genes (64.18%) were assigned a putative function (by cogs or by NR blast) and the ORFans are represented by 182 genes (6.04%). Finally, the rest of the genes were considered and annotated as hypothetical proteins (763 genes, 25.33%) (Table 4). Tables 5 and Table S1 summarize the properties and statistics of the genome. Figure S4 shows a graphical circular map of the genome and Figure 3 shows the distribution of functional classes of predicted genes on the chromosomes of strain CD2^T^. *Intestinimonas massiliensis* and its closest species seem for the most part associated with the same Clusters of Orthologous Groups (COG) genes. Nevertheless, COGs functional categories “Inorganic ion transport and metabolism, carbohydrate transport and metabolism, posttranslational modification, protein turnover, chaperones, cell wall/membrane biogenesis” are more represented in *Clostridium inocuum,* while that concerning “carbohydrate transport and metabolism” are more present in *Clostridium leptum*. In addition, *Pseudoflafonifractor capillosus* is enriched in genes belonging to COGs functional categories concerning “inorganic ion transport and metabolism, posttranslational modification, protein turnover, chaperones,” whereas COGs functional categories concerning “inorganic ion transport and metabolism” are more represented in *Flavonifractor plautii*.

**Table 4:** Nucleotide content and gene count levels of the genome

Attribute	Genome (total)	
	Value	% of total^a^
Size (bp)	3,104,261	100
G+C content (%)	1,882,912	60.66
Coding region (bp)	2,769,278	89.21
Total genes	3,074	100
RNA genes	62	2.02
Protein‐coding genes	3,012	100
Number of proteins associated with function prediction (nr+cogs not [S])	1,933	64.18
Number of proteins associated with hypothetical protein	763	25.33
Genes with function prediction	413	13.71
Genes assigned to COGs	134	4.45
Genes with peptide signals	375	12.45
Gene associated with resistance genes	1	0.03
Gene associated with bacteriocin genes	22	0.73
Proteins associated with ORFans	182	6.04
Genes associated with PKS or NRPS	9	0.29

a The total is based on either the size of the genome in base pairs or the total number of protein‐coding genes in the annotated genome.

**Table 5:** Pairwise comparison of *Intestinimonas massiliensis* GD2^T^ with other species using GGDC, formula 2 (DDH estimates based on identities/HSP length)* upper right. (1) *Intestinimonas massiliensis* GD2^T^; (2) *Pseudoflavonifractor capillosus* strain ATCC 29799; (3) *Flavonifractor plautii* strain Prevot S1; (4) *Intestinimonas butyriciproducens* strain SRB‐521‐5‐I; (5) *Clostridium viride* strain T2‐7; (6) *Oscillibacter valericigenes* strain Sjm18‐20; (7) *Sporobacter termitidis* strain SYR; (8) *Oscillibacter ruminantium* strain GH1; (9) *Butyricicoccus pullicaecorum* strain 25‐3

	1	2	3	4	5	6	7	8	9
1	100%	22.70 ± 2.4	21.70 ± 2.35	21.50 ± 2.35	26.40 ± 2.45	20.30 ± 2.30	18.40 ± 2.52	19.70 ± 2.30	28.10 ± 2.40
2		100%	22.20 ± 2.10	22.10 ± 2.35	20.50 ± 2.30	19.50 ± 2.30	19.40 ± 2.30	19.40 ± 2.30	25.00 ± 2.40
3			100%	22.00 ± 2.35	21.00 ± 2.35	21.10 ± 2.35	17.70 ± 2.25	19.00 ± 2.30	25.60 ± 2.40
4				100%	23.30 ± 2.40	20.40 ± 2.35	19.60 ± 2.30	20.30 ± 2.30	29.50 ± 2.45
5					100%	24.30 ± 2.40	24.20 ± 2.40	21.80 ± 2.35	24.60 ± 2.40
6						100%	22.20 ± 2.30	25.30 ± 2.40	26.90 ± 2.45
7							100%	26.10 ± 2.40	29.00 ± 2.40
8								100%	25.40 ± 2.45
9									100%

Confidence intervals indicate inherent uncertainty in estimating DDH values from intergenomic distances based on models derived from empirical test data sets (which are always limited in size).

### 3.6 Comparison of genome properties

The genome size, the G+C content, and the gene content of *I. massiliensis* and among the closest species are summarized in Table 2. In order to evaluate the genomic similarity among studied strains, we used two parameters: digital DDH (dDDH) that exhibits a high correlation with DDH (Auch, von Jan, Klenk, & Göker, 2010; Meier‐Kolthoff, Auch, Klenk, & Göker, 2013) and AGIOS (Ramasamy et al., 2014) that was designed to be independent from DDH. When considering only the closest species with standing in nomenclature for which a genome is available, dDDH values ranged from 17.70 ± 2.25 between *Flavonifractor plautii* and *Sporobacter termitidis* to 29.50 ± 2.45 between *Intestinimonas butyriciproducens* and *Butyricicoccus pullicaecorum*. When we include the strain GD2^T^ in the comparison, the dDDH values ranged from 18.40 ± 2.52 with *Sporobacter termitidis* to 28.10 ± 2.40 with *Butyricicoccus pullicaecorum* (Table 5).

Regarding AGIOS, values ranged from 52.31 between *Pseudoflavonifractor capillosus* and *Clostridium cellulosi* to 73.57% between *Flavonifractor plautii* and *Intestinimonas butyriciproducens* among compared species. Including *Intestinimonas massiliensis*, AGIOS ranged from 57.10 with *Ethanoligenens harbinense* to 76.46% with *Intestinimonas butyriciproducens* (Table S2). As the obtained dDDH values were lower than 70%, and because dDDH and AGIOS values were close to the range of those obtained among compared species with standing in nomenclature, and because of the production of butyrate and acetate, and finally because the difference of G+C content with other *Intestinimonas* species was greater than 1 with *Intestinimonas butyriciproducens* (Table 2) (Meier‐Kolthoff, Klenk, & Göker, 2014), we are confident that strain GD2^T^ is the representative strain of a new species within the genus *Intestinimonas*.

### 3.7 Frequency and relative abundance of *I. massiliensis* and *I. butyriciproducens*

With a similarity threshold of 99%, *I. massiliensis* was detected in 4.40% of all datasets, mainly involving the human gut, but also in animals and the environment. In comparison, *I. butyriciproducens* was present in 1.98% of all available 16S rRNA amplicon datasets. Interestingly, *I. massiliensis* was detected more frequently than *I. butyriciproducens* in the human gut, as they were present in 19.8% of and in 8.1% of the 16,950 datasets, respectively (Chi‐squared test <10^−7^) (Table S3). The mean relative abundances from these datasets were of 0.079% and 0.087% for *I. massiliensis and I. butyriciproducens*, respectively.

## 4 DISCUSSION

Herein, we describe a new species belonging to the genus *Intestinimonas*. The strain GD2^T^ was isolated for the first time in the stool of a healthy 28‐year‐old French male using a “culturomics” approach. Based on different biochemical, phylogenetic, and genomic properties when compared with the phylogenetically closest species (i.e., *Intestinimonas butyriciproducens* SRB‐521‐5‐I^T^) (Fournier et al., 2015), we proposed the creation of the second bacterial species, strain GD2^T^, belonging to the genus *Intestinimonas*.

Like Kläring et al. (2013) with *I*.* butyriciproducens*, we experienced difficulties in determining if the strain GD2^T^ was gram positive or negative. Indeed, gram staining combined with optical microscopy revealed the presence of gram‐negative bacilli. In addition, the susceptibility to vancomycin as well as its classification with gram‐positive microbes led us to assume that *I. massiliensis* should be considered as a gram‐positive microorganism, according to the genus formal description (Kläring et al., 2013). However, we did not observe by transmission electron microscopy (in ultrathin sections of resin‐embedded cells) a clear membrane arrangement of the cells resembling a gram+ ultrastructure (Figure S5).

*Intestinimonas massiliensis* significantly produces butyrate, which is an SCFA of potential medical importance. Butyrate is known to be an energy source for epithelial cells and plays a key role in maintaining homeostasis of colonic cells. In addition, several works have shown its inhibiting role in inflammation and oxidative stress (Hamer et al., 2008), whereas its contribution to improving insulin sensitivity and glucose homeostasis has been reported, as with other SCFAs (Canfora, Jocken, & Blaak, 2015).

Also, being detected more frequently in 16S rRNA amplicon datasets than *Intestinimonas butyriciproducens*,* Intestinimonas massiliensis* appears to be a common human gut commensal that may contribute to the gut microbiota homeostasis.

## 5 CONCLUSION

With a similarity level of 94.96% to the strain *Intestinimonas butyriciproducens* gen. nov., sp. nov and based on phenotypic, genomic, and phylogenetic characteristics, we have isolated a new species, named *Intestinimonas massiliensis* sp. nov strain GD2^T^, isolated for the first time in the human gut microbiota. The 16S rRNA gene sequence and whole‐genome shotgun sequence of *Intestinimonas massiliensis* strain GD2^T^ has been deposited in GenBank with the accession Number LN866996.

## 6 DESCRIPTION OF *INTESTINIMONAS MASSILIENSIS* SP. NOV STRAIN GD2^T^ (= CSUR P1930, = DSM100417)

### 6.1 *Intestinimonas massiliensis* (mas.si.li.en'sis. L. masc. adj. *massiliensis* of *Massilia*, the ancient Roman name for Marseille, where the strain was isolated)

Strictly anaerobic, gram‐negative, oxidase and catalase negative, nonendospore forming, and nonmotile rods, the colonies are circular, small, and glossy with a diameter of approximately 0.5–1 mm on Columbia agar + 5% sheep blood. Growth was noticed at 37°C after 3–4 days of incubation and with a pH between 6 and 8.5. Cells measure about 1–1.5 μm in length and 0.5 μm in diameter.

Using API 50 CH and ZYM strips, positive reactions were observed for: arabinose, D‐ribose, D‐xylose, L‐xylose, D‐galactose, D‐fructose, L‐sorbose, amygdalin, esculin ferric citrate, D‐melibiose, D‐trehalose, inulin, D‐melezitose, D‐raffinose, starch, glycogen, xylitol, gentiobiose, D‐lyxose, D‐tagatose, D‐fucose, potassium 5‐ketogluconate, naphthol‐AS‐BI‐phosphohydrolase, and acid phosphatase. The API 20 NE strip showed a positive reaction for gelatin hydrolysis and negative reaction for other biochemical tests. *Intestinimonas massiliensis* sp. nov strain GD2^T^ is susceptible to amoxicillin, ceftriaxone, penicillin G, imipenem, and vancomycin. With regard to fatty acids, an abundance of unsaturated 9‐octadecenoic acid (35%) and saturated hexadecanoic acid (30%) was observed. This bacterium produces acetic (0.7 ± 0.1 mmol/L), propanoic (0.4 ± 0.4 mmol/L), butanoic (6.4 ± 0.7 mmol/L), and pentanoic (0.1 ± 0.1 mmol/L) acids.

The G+C content of the genome is 60.68%. Accession numbers of the sequences of 16S rRNA and genome deposited in EMBL‐EBI are LN866996 and CWJP00000000, respectively. The microorganism was isolated within the human gut microbiota. The type strain GD2^T^ (= CSUR P1930 =  DSM100417) was isolated from a stool specimen of a healthy 28‐year‐old French male.

## CONFLICT OF INTEREST

The authors declare no financial conflict of interest.
