# EXPRESSION OF CONCERN: Noncontiguous Finished Genome Sequence and Description of *Intestinimonas massiliensis* sp. nov Strain GD2T, the Second *Intestinimonas* Species Cultured from the Human Gut

**DOI:** 10.1002/mbo3.70249

**Published:** 2026-02-19

## Abstract

**EXPRESSION OF CONCERN:** P. Afouda, G.A. Durand, J.‐C. Lagier, N. Labas, F. Cadoret, N. Armstrong, D. Raoult, and G. Dubourg, “Noncontiguous Finished Genome Sequence and Description of *Intestinimonas massiliensis* sp. nov Strain GD2T, the Second *Intestinimonas* Species Cultured from the Human Gut,” *Microbiology Open* 8, no. 1 (2019): e00621, https://doi.org/10.1002/mbo3.621.

This Expression of Concern is for the above article, published online on 14 April 2018 in Wiley Online Library (wileyonlinelibrary.com), and has been issued by John Wiley & Sons Ltd. The Expression of Concern has been agreed due to questions raised about the study's adherence to French legal and ethical requirements for research involving human subjects. The investigation into these concerns is ongoing. Therefore, the journal has decided to issue an Expression of Concern to inform and alert readers.

