# Supplementary material for: Noncontiguous finished genome sequence and description of Intestinimonas massiliensis sp. nov strain GD2T, the second Intestinimonas species cultured from the human gut
Source: Microbiologyopen. 2018 Apr 14;8(1):e00621. doi: 10.1002/mbo3.621 (PMC6341035; doi:10.1002/mbo3.621)
Supplement: Supplementary file 1 [file MBO3-8-e00621-s001.docx]

Supplementary Data

**Non-contiguous finished genome sequence and description of *Intestinimonas massilien*sis sp. nov strain GD2^T^, the second *Intestinimonas* species cultured from the human gut**

Pamela AFOUDA^1^, Guillaume A. DURAND^1^, Jean-Christophe LAGIER^1^, Noémie LABAS^1^, Fréderic CADORET^1^, Nicholas ARMSTRONG ^1^, Didier RAOULT^1^ and Grégory DUBOURG^1^*

*Corresponding author : Dr Grégory DUBOURG : greg.dubourg@gmail.com

1. **Supplementary figures**


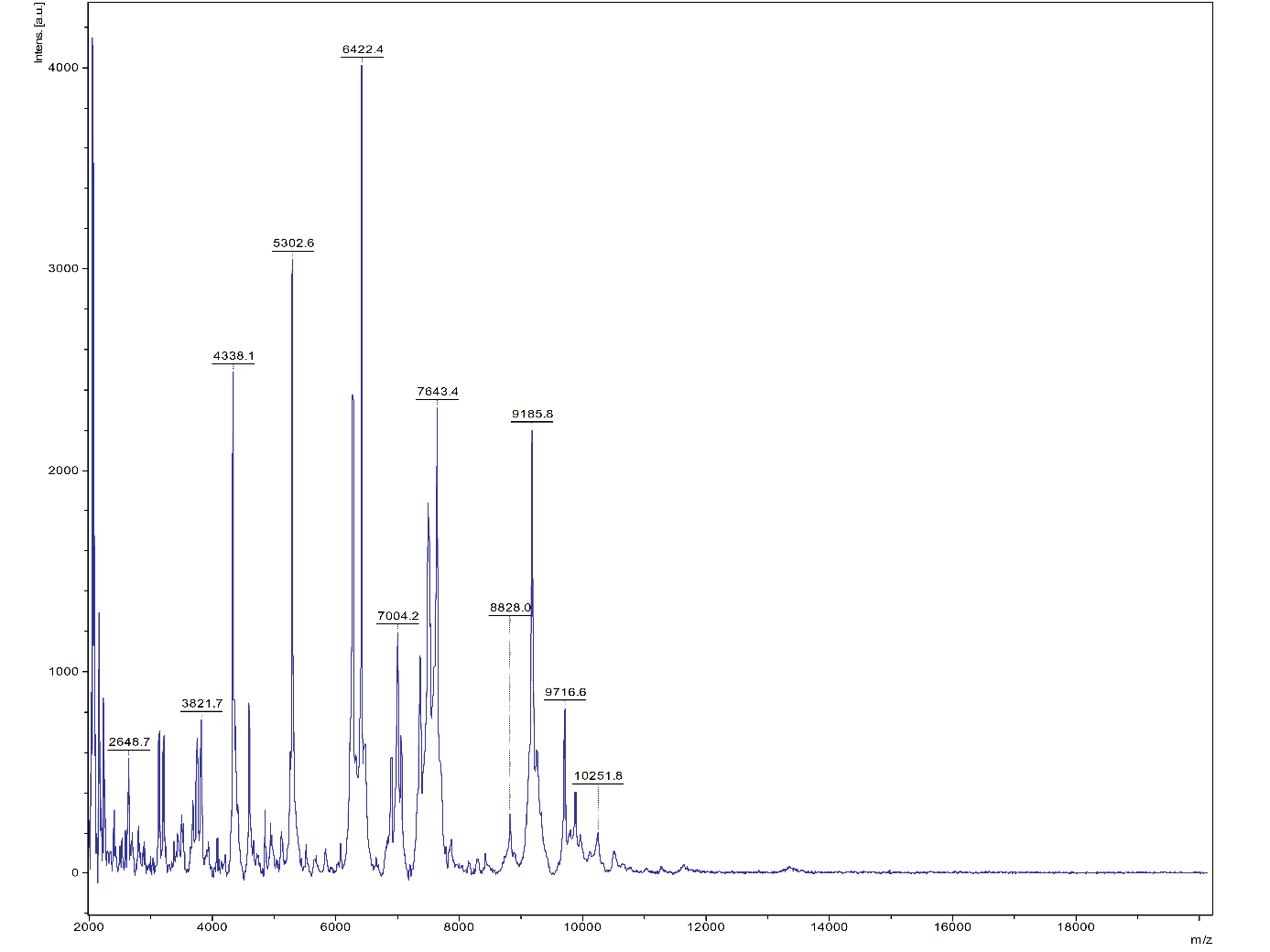


**Figure S1.** Spectrum of *Intestinimonas massiliensis* strain GD2^T^ obtained on MALDI-TOF Spectra from 16 individual colonies were compared and a reference spectrum was generated. FlexAnalysis Software (Bruker Daltonics, Bremen, Germany) was used for visual inspection and mass spectra processing such as smoothing, baseline subtraction, and peak picking. The mass range from m / z 2,000 to 20,000 is reprensented on the x-axis and the y-axis represents the peak intensity (in arbitrary units).


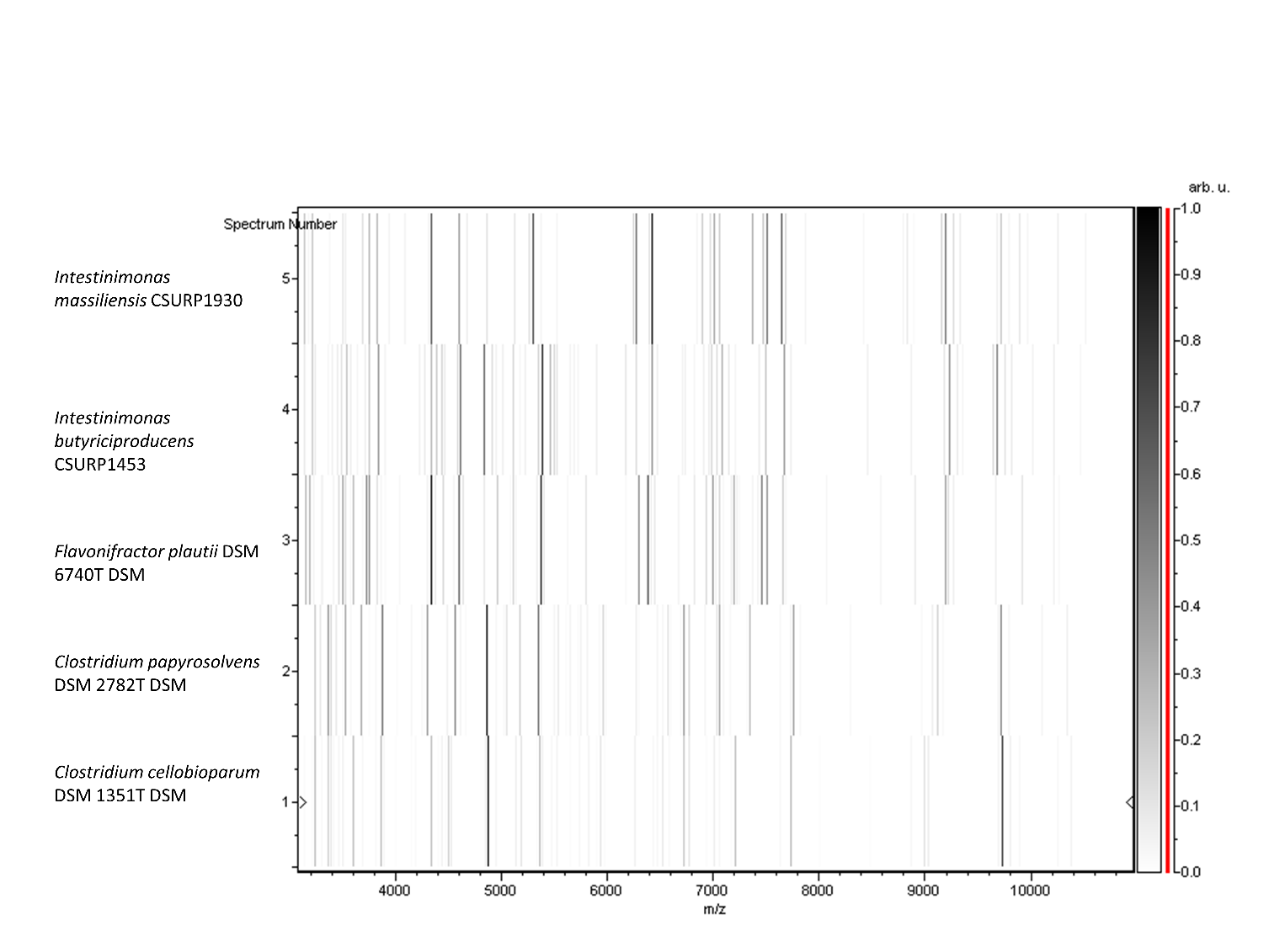


**Figure S2.** Gel View *Intestinimonas massiliensis* strain GD2^T^ relative to other Firmicutes. The gel view displays the raw spectra of loaded spectrum files arranged in a pseudo gel-like look. The x-axis records the m/z value. The left y-axis displays the running spectrum number originating from subsequent spectra loading. The peak intensity is expressed by a grayscale code. The color bar and the right y-axis indicate the relation between the color with which a peak is displayed and the peak intensity in arbitrary units.


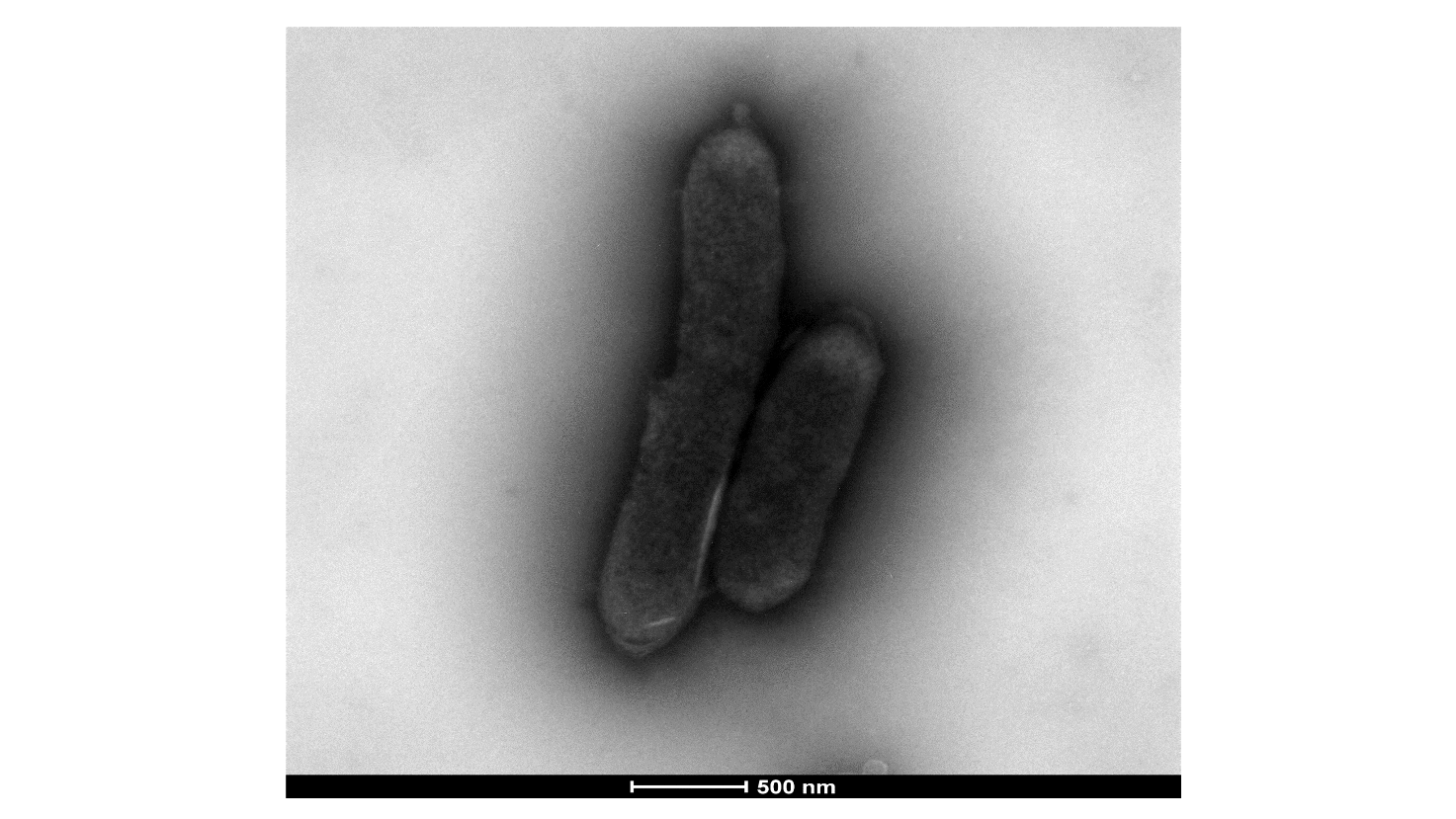


**Figure S3.** Transmission electron microscopy of *Intestinimonas massiliensis* strain GD2^T^, using a Tecnai G20 (FEI company). The scale bar represents 500 nm


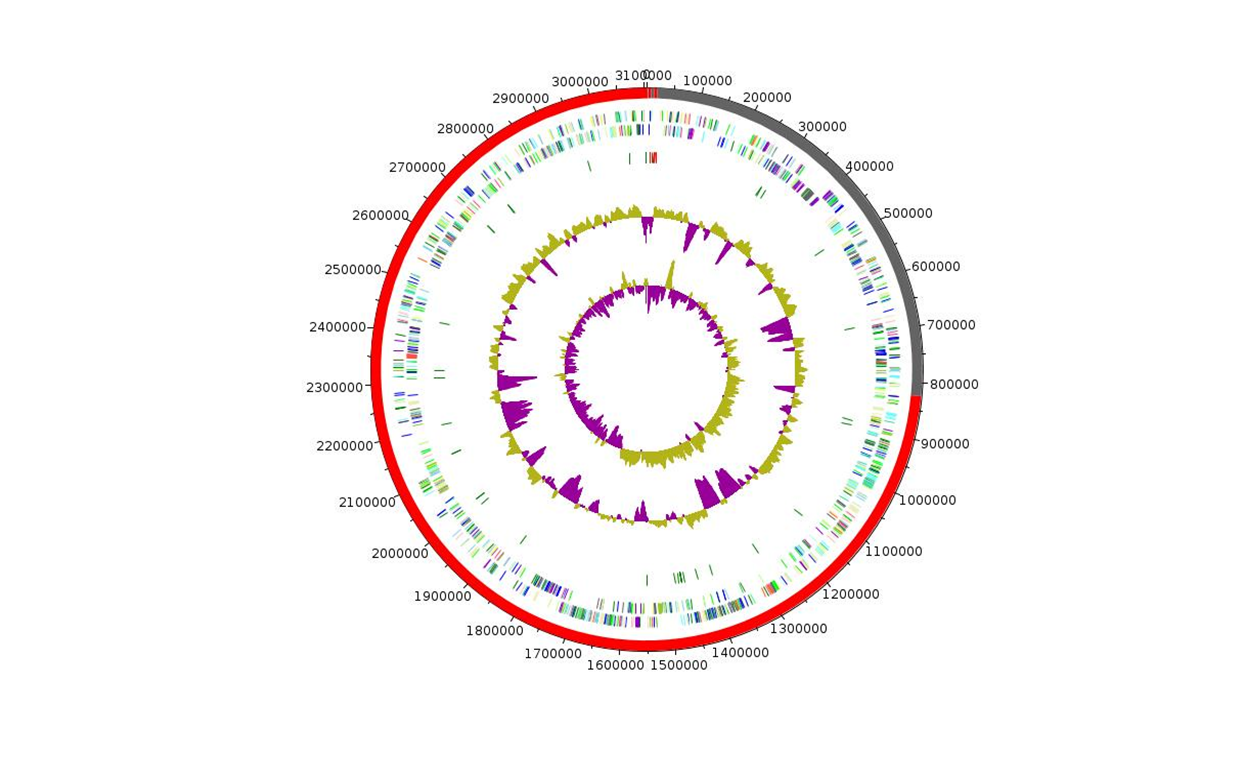


**Figure S4.** Graphical circular map of the genome of strain CD2^T^. From outside to the center: Contigs (red/grey), COG category of genes on the forward strand (three circles), genes on forward strand (blue circle), genes on the reverse strand (red circle), COG category on the reverse strand (three circles), GC content.


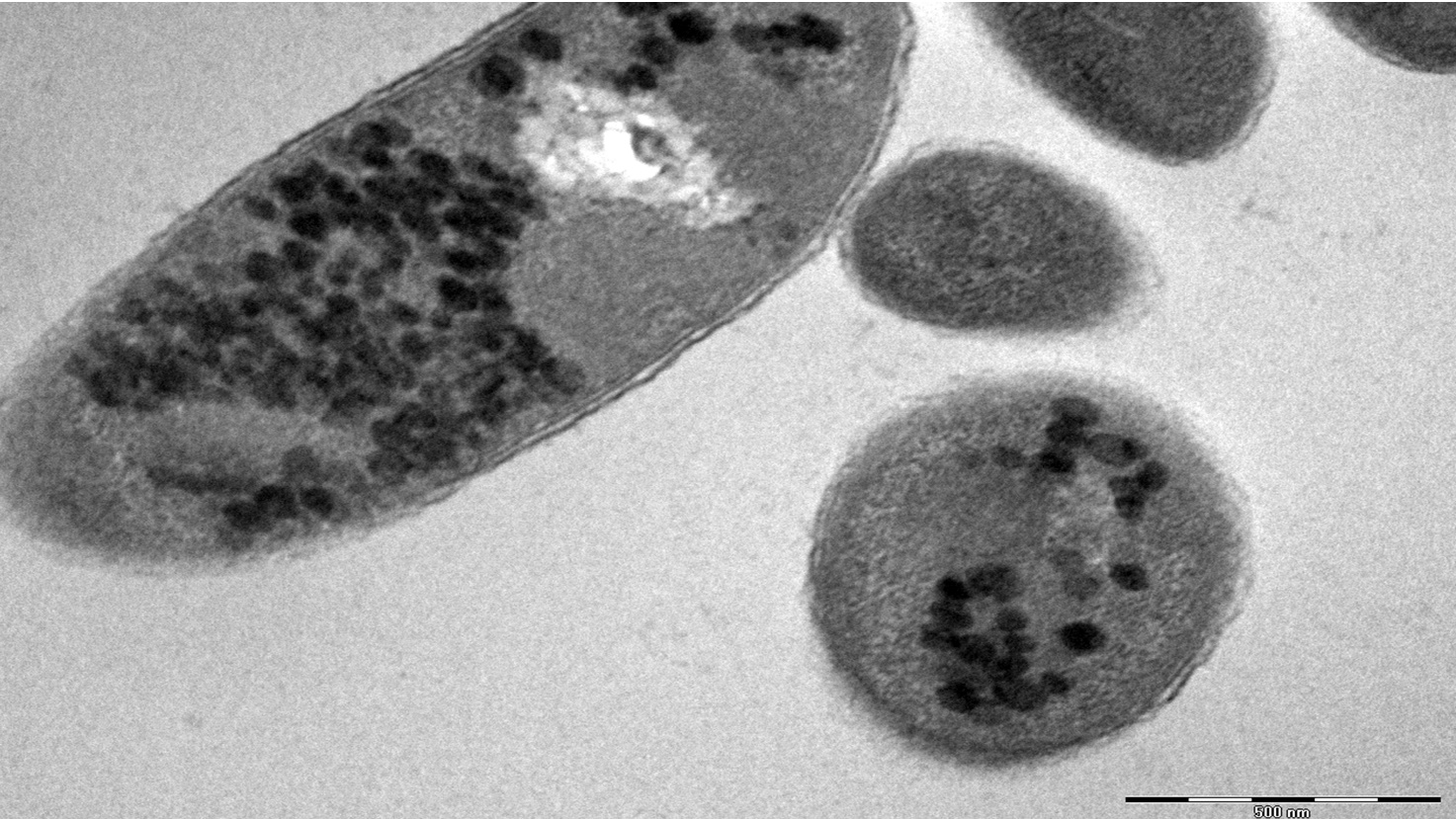


**Figure S5**. Transmission electron microscopy of *Intestinimonas massiliensis* strain GD2^T^, using a Morgagni 268D TEM (Philips) for evaluating elements attesting to the Gram type. The scale bar represents 500 nm.

1. **Supplementary Table**

| **Code** | **Value** | **% of total**^a^ | **Description** |
| --- | --- | --- | --- |
| J | 147 | 4.88 | Translation |
| A | 0 | 0 | RNA processing and modification |
| K | 173 | 5.74 | Transcription |
| L | 129 | 4.28 | Replication, recombination and repair |
| B | 0 | 0 | Chromatin structure and dynamics |
| D | 20 | 0.66 | Cell cycle control, mitosis and meiosis |
| Y | 0 | 0 | Nuclear structure |
| V | 45 | 1.49 | Defense mechanisms |
| T | 63 | 2.09 | Signal transduction mechanisms |
| M | 60 | 1.99 | Cell wall/membrane biogenesis |
| N | 1 | 0.03 | Cell motility |
| Z | 0 | 0 | Cytoskeleton |
| W | 0 | 0 | Extracellular structures |
| U | 20 | 0.66 | Intracellular trafficking and secretion |
| O | 54 | 1.79 | Post-translational modification, protein turnover, chaperones |
| C | 116 | 3.85 | Energy production and conversion |
| G | 110 | 3.65 | Carbohydrate transport and metabolism |
| E | 214 | 7.10 | Amino acid transport and metabolism |
| F | 68 | 2.26 | Nucleotide transport and metabolism |
| H | 46 | 1.53 | Coenzyme transport and metabolism |
| I | 81 | 2.69 | Lipid transport and metabolism |
| P | 106 | 3.52 | Inorganic ion transport and metabolism |
| Q | 29 | 0.96 | Secondary metabolites biosynthesis, transport and catabolism |
| R | 202 | 6.71 | General function prediction only |
| S | 134 | 4.45 | Function unknown |
| - | 1358 | 45.09 | Not in COGs |

^a^ The total is based on the total number of protein coding genes in the annotated genome

**Table S1.** Number of genes associated with the 25 general COG functional categories

|  | *Intestinimonas butyriciproducens* | *Flavonifractor plautii* | *Clostridium leptum* | *Intestinimonas massiliensis* | *Clostridium cellulosi* | *Ethanoligenens harbinense* | *Oscillibacter valericigenes* | *Clostridium viride* | *Eubacterium siraeum* | *Pseudoflavonifractor capillosus* |
| --- | --- | --- | --- | --- | --- | --- | --- | --- | --- | --- |
| *Intestinimonas butyriciproducens* | **3529** | 1120 | 512 | 1071 | 622 | 494 | 781 | 675 | 409 | 1016 |
| *Flavonifractor plautii* | 73.57 | **4278** | 525 | 1002 | 636 | 510 | 806 | 686 | 424 | 1025 |
| *Clostridium leptum* | 61.63 | 61.67 | **2482** | 509 | 583 | 499 | 479 | 433 | 407 | 528 |
| *Intestinimonas massiliensis* | 76.46 | 74.07 | 61.93 | **3012** | 595 | 496 | 789 | 673 | 398 | 946 |
| *Clostridium cellulosi* | 58.01 | 56.92 | 61.74 | 57.54 | **5171** | 612 | 600 | 564 | 453 | 606 |
| *Ethanoligenens harbinense* | 56.86 | 56.79 | 56.06 | 57.10 | 55.25 | **2701** | 510 | 445 | 406 | 490 |
| *Oscillibacter valericigenes* | 65.20 | 65.46 | 61.50 | 65.98 | 58.45 | 56.38 | **4723** | 658 | 408 | 709 |
| *Clostridium viride* | 64.47 | 64.18 | 60.79 | 64.44 | 59.21 | 54.97 | 62.59 | **2321** | 368 | 615 |
| *Eubacterium siraeum* | 58.90 | 57.65 | 60.68 | 59.10 | 61.46 | 54.35 | 58.90 | 58.92 | **2211** | 424 |
| *Pseudoflavonifractor capillosus* | 61.79 | 62.94 | 55.51 | 62.15 | 52.31 | 57.23 | 58.16 | 56.72 | 53.14 | **4829** |

**Table S2.** Orthologous proteins shared between genomes (upper right); average percentage similarity of nucleotides corresponding to orthologous proteins shared between genomes (lower left) and numbers of proteins per genome (bold).

| **Bacteria** | **Origin of 16S rRNA gene datasets** | **N positive datasets** | **N of datasets analyzed** | **Frequency** | **Mean relative abundance** | **Median relative abundance** |
| --- | --- | --- | --- | --- | --- | --- |
| *Intestinomonas massiliensis* GD2^T^ | All | 4263 | 96869 | 4,40% |  |  |
|  | Human gut | 3356 | 16950 | 19,80% | 0,079% | 0,046% |
| *Intestinimonas butyriciproducens*  SRB-521-5-I^T^ |  |  |  |  |  |  |
|  | All | 1914 | 96869 | 1,98% |  |  |
|  | Human gut | 1373 | 16950 | 8,10% | 0,088% | 0,031% |
|  |  |  |  |  |  |  |

**Table S3.** Frequency and relative abundance of *Intestinomonas massiliensis* GD2^T^ and *Intestinimonas butyriciproducens* SRB-521-5-I^T^ among 16S rRNA gene datasets available in the Sequence Read Archive using IMNGS plateform (39).
